# POC CD4 Testing Improves Linkage to HIV Care and Timeliness of ART Initiation in a Public Health Approach: A Systematic Review and Meta-Analysis

**DOI:** 10.1371/journal.pone.0155256

**Published:** 2016-05-13

**Authors:** Lara Vojnov, Jessica Markby, Caroline Boeke, Lindsay Harris, Nathan Ford, Trevor Peter

**Affiliations:** 1 Clinton Health Access Initiative, Boston, MA, United States of America; 2 World Health Organization, Geneva, Switzerland; CEA, FRANCE

## Abstract

**Background:**

CD4 cell count is an important test in HIV programs for baseline risk assessment, monitoring of ART where viral load is not available, and, in many settings, antiretroviral therapy (ART) initiation decisions. However, access to CD4 testing is limited, in part due to the centralized conventional laboratory network. Point of care (POC) CD4 testing has the potential to address some of the challenges of centralized CD4 testing and delays in delivery of timely testing and ART initiation. We conducted a systematic review and meta-analysis to identify the extent to which POC improves linkages to HIV care and timeliness of ART initiation.

**Methods:**

We searched two databases and four conference sites between January 2005 and April 2015 for studies reporting test turnaround times, proportion of results returned, and retention associated with the use of point-of-care CD4. Random effects models were used to estimate pooled risk ratios, pooled proportions, and 95% confidence intervals.

**Results:**

We identified 30 eligible studies, most of which were completed in Africa. Test turnaround times were reduced with the use of POC CD4. The time from HIV diagnosis to CD4 test was reduced from 10.5 days with conventional laboratory-based testing to 0.1 days with POC CD4 testing. Retention along several steps of the treatment initiation cascade was significantly higher with POC CD4 testing, notably from HIV testing to CD4 testing, receipt of results, and pre-CD4 test retention (all p<0.001). Furthermore, retention between CD4 testing and ART initiation increased with POC CD4 testing compared to conventional laboratory-based testing (p = 0.01). We also carried out a non-systematic review of the literature observing that POC CD4 increased the projected life expectancy, was cost-effective, and acceptable.

**Conclusions:**

POC CD4 technologies reduce the time and increase patient retention along the testing and treatment cascade compared to conventional laboratory-based testing. POC CD4 is, therefore, a useful tool to perform CD4 testing and expedite result delivery.

## Introduction

Many HIV patients currently do not have reliable access to essential diagnostic laboratory tests, including CD4, clinical chemistry, hematology, viral load, and diagnosis of common co-infections. CD4 testing is critical for identifying patients most in need of clinical care and antiretroviral therapy (ART) [1–4]. While there is a progressive move away from CD4 cell count as the main way to determine eligibility for ART [5], CD4 cell count testing still has an important role to play in baseline risk assessment, prioritizing patients when limited ART drug supplies exist, and diagnosing treatment failure in settings where access to viral load monitoring is limited [6,7]. Furthermore, CD4 testing remains beneficial for HIV disease and opportunistic infection management of patients on ART.

Conventional laboratory-based CD4 testing presents several key challenges including use of complicated equipment that is poorly adapted for resource-limited settings, requires un-interrupted power supply, cold chain logistics for reagents, regular technical support and maintenance, and a high skill level for operation as well as large capital outlay for equipment. Due to these constraints, conventional CD4 testing is limited to higher tiered laboratory settings that often limit the access to testing. Transport of whole blood samples to centralized laboratories and return of results to clinical sites is a challenge in resource-limited settings often resulting in invalid specimens, inaccurate results, long turn around times for results or lost results. Delays caused by conventional testing and result delivery can lead to patients being lost before ART initiation [8].

Several studies have highlighted the need to improve patient retention prior to ART initiation in the current cascade of care [8–11]. Just over half of known HIV-positive patients receive a CD4 test result, while less than three-quarters of patients determined eligible are initiated on ART [9–11]. Pre-ART retention has been substantially low at under 50% in previous meta-analyses [9,11]. The requirement of multiple visits for sample collection, results received, and clinical decision-making can lead to increased transportation costs and distances, long waiting times, and the requirement to take time away from work, all of which are associated with loss [8].

POC diagnostic technologies may alleviate some of these burdens on the health care system by decentralizing care and testing, providing immediate test results, and allowing for expedited clinical decisions. Several POC CD4 technologies are currently on the market [12] and several are in the development pipeline. POC CD4 technologies can reduce the number of trips to the health care facility required of patients in the pre-ART staging process, thus decreasing costs, effort and time away from work. Same day HIV diagnosis, CD4 testing, and ART eligibility assessments can lower the pre-ART attrition observed in most high HIV burden settings [13].

A previous systematic review published in 2013 reported improved retention along the testing and treatment cascade with the introduction and use of POC CD4 technologies [14]; however, this review was conducted early in the adoption of POC CD4 and the number of studies contributing to the review was small and limited in the number of outcomes it was able to assess. We conducted this systematic review and meta-analysis to update the patient impact of POC CD4 compared to conventional laboratory-based testing to support the 2016 revision of the World Health Organization (WHO) guidelines for the treatment and care of people living with HIV.

## Methods

### Search strategy and study selection

Standard global guidelines were followed for the search strategy and study selection including the Preferred Reporting Items for Systematic Reviews and Meta-Analyses (PRISMA) [15] (S1 Fig). PubMed and EMBASE databases were searched from 1 January 2005 to 15 April 2015. Search terms were developed using the PICO question format (population, intervention, comparator, outcome). The search terms were, therefore, broken into four categories: HIV (population), POC (intervention), CD4 (intervention/comparator), and outcomes. Characteristics of POC technologies as well as manufacturer names were included to maximize study identification as well as an extensive list of outcomes (S2 Fig). Conference abstracts within the search dates from the Conference on Retroviruses and Opportunistic Infections (CROI), International Conference on AIDS and STIs in Africa (ICASA), International AIDS Society (IAS), and AIDS Conference and bibliographies were also screened and reviewed for possible inclusion. Search terms included characteristics of POC technologies, outcome terms, and manufacturer names to maximize study identification. Two independent reviewers screened all titles and abstracts for eligibility. Studies were included if they compared the impact of POC to laboratory-based CD4 testing in HIV-positive patients. No geographical region or age restrictions were applied. Only English language studies were considered for inclusion. Data was extracted from each study included: the POC CD4 technology used, sample size, test setting, study dates, and key outcomes as defined in the study protocol. Studies were assessed for quality, bias and applicability using the QUADAS-2 guidelines [16].

### Data analysis

The primary outcomes of interest were time and retention along the testing and treatment cascade. Secondary outcomes included cost-effectiveness and acceptability. To determine the presence of between-study heterogeneity, the Q-statistic was calculated [17]. Random effects models were used to estimate the pooled summary measures for turnaround time, proportions of retention, risk ratios, and 95% confidence intervals. For the random effects model of proportions, the metaprop command was used in Stata with a continuity correction value of 0.5 and exact confidence intervals. To calculate risk ratios, the metan command was used, specifying use of the DerSimonian and Laird method using the inverse-variance fixed effect model for the estimate of heterogeneity. Weighted averages were used to compare continuous outcomes for patients tested with a POC technology compared to those tested with a conventional laboratory-based technology.

Two researchers independently performed the statistical analysis to ensure accuracy. Graphic representations were completed in GraphPad Prism v6.0 (La Jolla, California, USA) and analyses were completed in Stata 13 (College Station, Texas, USA).

## Results

### Study characteristics and assessment

**Fig 1:**
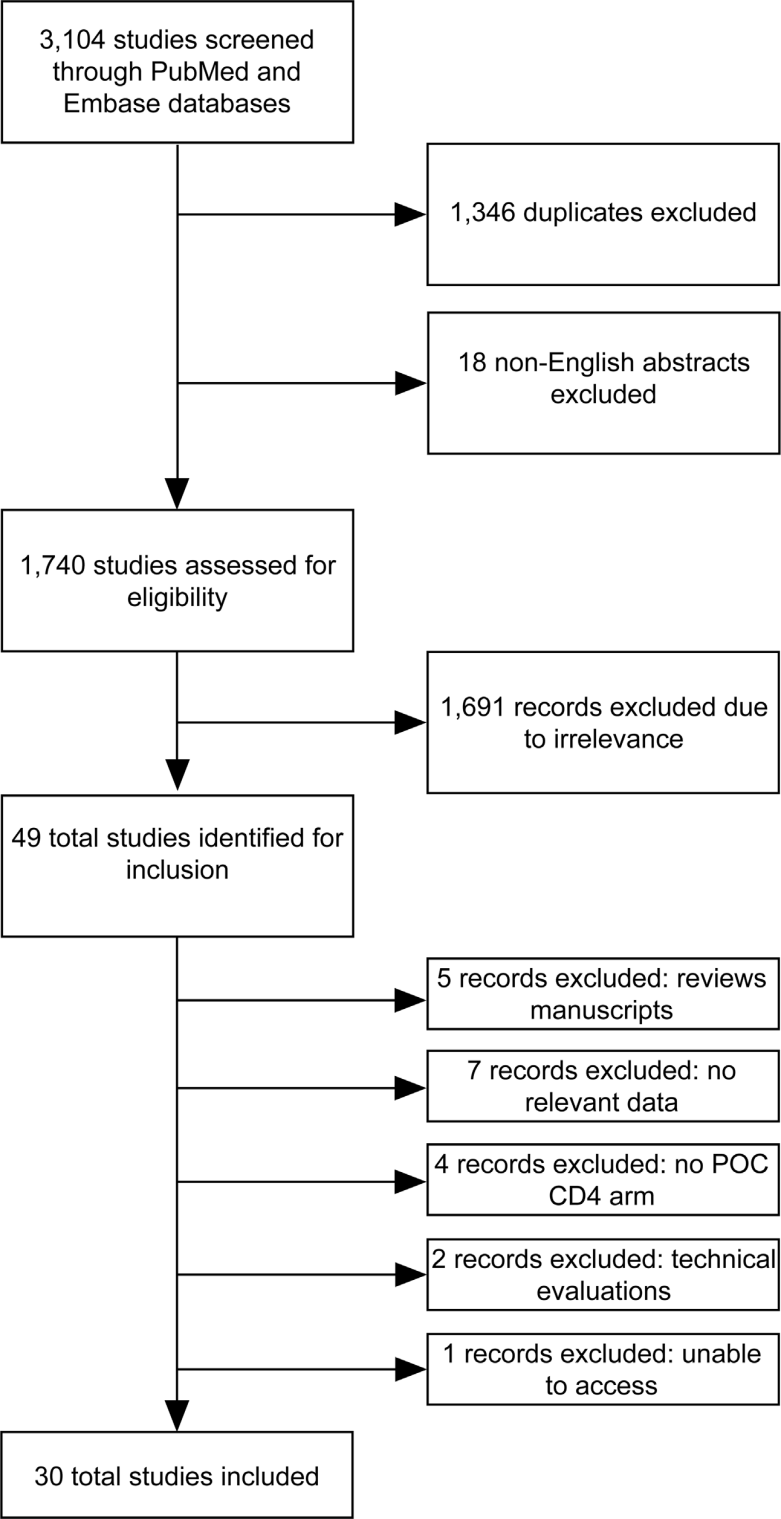
PRISMA diagram of search outcome and included studies.

The search strategy yielded 30 studies relevant to the study question (Fig 1 and Table 1) [13,18–46]. Almost 40,000 total patients were included in the analysis, while the median sample size per study was 830 HIV-positive patients. All studies included adults; however, one study focused on youth between 12 and 25 years of age. Five studies included pregnant women as the primary population. The included studies spanned eight countries, most within sub-Saharan Africa. All studies were conducted between 2009 and 2015. Two POC CD4 technologies were included with 25 of 27 studies using the Alere Pima POC CD4. There were three randomized control trials, four prospective observational studies, 17 retrospective observational studies, and six cost-effectiveness or costing studies.

**Table 1:** Study characteristics.

#	Author	Journal	Year	Countries of study	Type of study	Years of study	Site type	Population	POC technology	# of participants
1	Barnabas	Lancet	2014	South Africa, Uganda	prospective observational	2011–2013	home-based	adults	Alere Pima	3545
2	Bassett	PLoS One	2014	South Africa	cost-effectiveness	2010–2011	mobile clinic	adults	Alere Pima	NA
3	Brown	IAS poster	2013	Tanzania	retrospective observational	2011–2012	clinics	adults	Alere Pima	NI
4	Cassim	PLoS One	2014	South Africa	costing	2012–2013	NA	adults	Alere Pima	NA
5	Chien	IAS poster	2013	Uganda	retrospective observational	2011–2012	clinics	adults	Alere Pima	NI
6	Ciaranello	PLoS One	2015	South Africa	cost-effectiveness	NA	NA	infants, pregnant women	generic	NA
7	De Schacht	IAS abstract	2012	Mozambique	retrospective observational	2010–2011	EGPAF clinics	pregnant adults	Alere Pima	3410
8	Desai	CROI poster	2015	Kenya	prospective RCT	2013–2014	home-based/clinics	adults	Alere Pima	770
9	Dhoot	IJSA	2013	UK	retrospective observational	2011–2012	NI	adults	Alere Pima	44
10	Faal	JAIDS	2011	South Africa	prospective RCT	2009	clinic	adults	BD FACSCount	344
11	Fajardo	AIDS poster	2014	9 sSA	retrospective observational	2011–2013	MSF clinics	adults	Alere Pima	25749
12	Grundy	presentation	unpublished	NA	cost-effectiveness	NI	NI	NA	generic	NA
13	Hatzold	IAS abstract	2011	Zimbabwe	retrospective observational	NI	PSI clinics	adults	not indicated	182
14	Herbert	HIV Medicine	2011	UK	retrospective observational	2010–2011	clinic	adults	Alere Pima	200
15	Hyle	PLoS One	2014	Mozambique	cost-effectiveness	NA	NA	adults	Alere Pima	NA
16	Jani	Lancet	2011	Mozambique	retrospective observational	2009–2010	clinics	adults	Alere Pima	1021
17	Jani	AIDS	2015	Mozambique	retrospective observational	2013–2014	clinics	adults	Alere Pima	103795
18	Larson	PLoS One	2012	South Africa	costing	NA	mobile clinic	adults	Alere Pima	NA
19	Larson	JAIDS	2012	South Africa	retrospective observational	2010–2011	mobile clinic	adults	Alere Pima	508
20	Larson	AIDS Res Tre	2013	South Africa	retrospective observational	2008–2010	clinic	adults	Alere Pima	897
21	Larson	IAS abstract	2011	South Africa	retrospective observational	2010	clinic	adults	BD FACSCount	538
22	Muchedzi	IAS abstract	2012	Zimbabwe	retrospective observational	2011	EGPAF clinics	pregnant adults	Alere Pima	2310
23	Myer	JAIDS	2015	South Africa	prospective observational	2010–2013	clinic	pregnant adults	Alere Pima	19432
24	Myer	AIDS Care	2012	South Africa	retrospective observational	2011	clinic	pregnant adults	Alere Pima	2290
25	Obi	unclear	2013	UK	prospective observational	NI	hospital	adults	Alere Pima	199
26	Patten	JIAS	2013	South Africa	retrospective observational	2010–2012	clinics	youth, 12-25y	Alere Pima	576
27	Rioja	IAS poster	2013	Cameroon	retrospective observational	2012	hospitals	adults	Alere Pima	NI
28	Rosen	CROI poster	2015	South Africa	prospective RCT	2013–2014	clinics	adults	Alere Pima	598
29	Tsibolane	in preparation	2014	South Africa	retrospective observational	2014	clinics	adults	Alere Pima	1492
30	van Rooyen	JAIDS	2013	South Africa	prospective observational	2011–2012	home-based	adults	Alere Pima	671

NI: not indicated

NA: not applicable

Overall, there was a moderate risk of bias due to potential confounding temporal, facility- or individual-level factors. Most studies were observational cross-sectional studies. Participant samples in most studies were not consecutively recruited or studies failed to report the process of patient recruitment (70%). The rationale for facility selection was not indicated or not randomized in most studies (72%). Some studies used a pre-post analysis while others compared different groups of facilities with and without POC CD4 devices. There were some potential limitations in geographical applicability: most (90%) studies were carried out in Africa in field settings and 50% were carried out in one country (South Africa). Additionally, most studies (78%) did not include POC CD4 testing using finger-prick samples or failed to report the specimen type.

### Timeliness of testing and ART initiation

**Fig 2:**
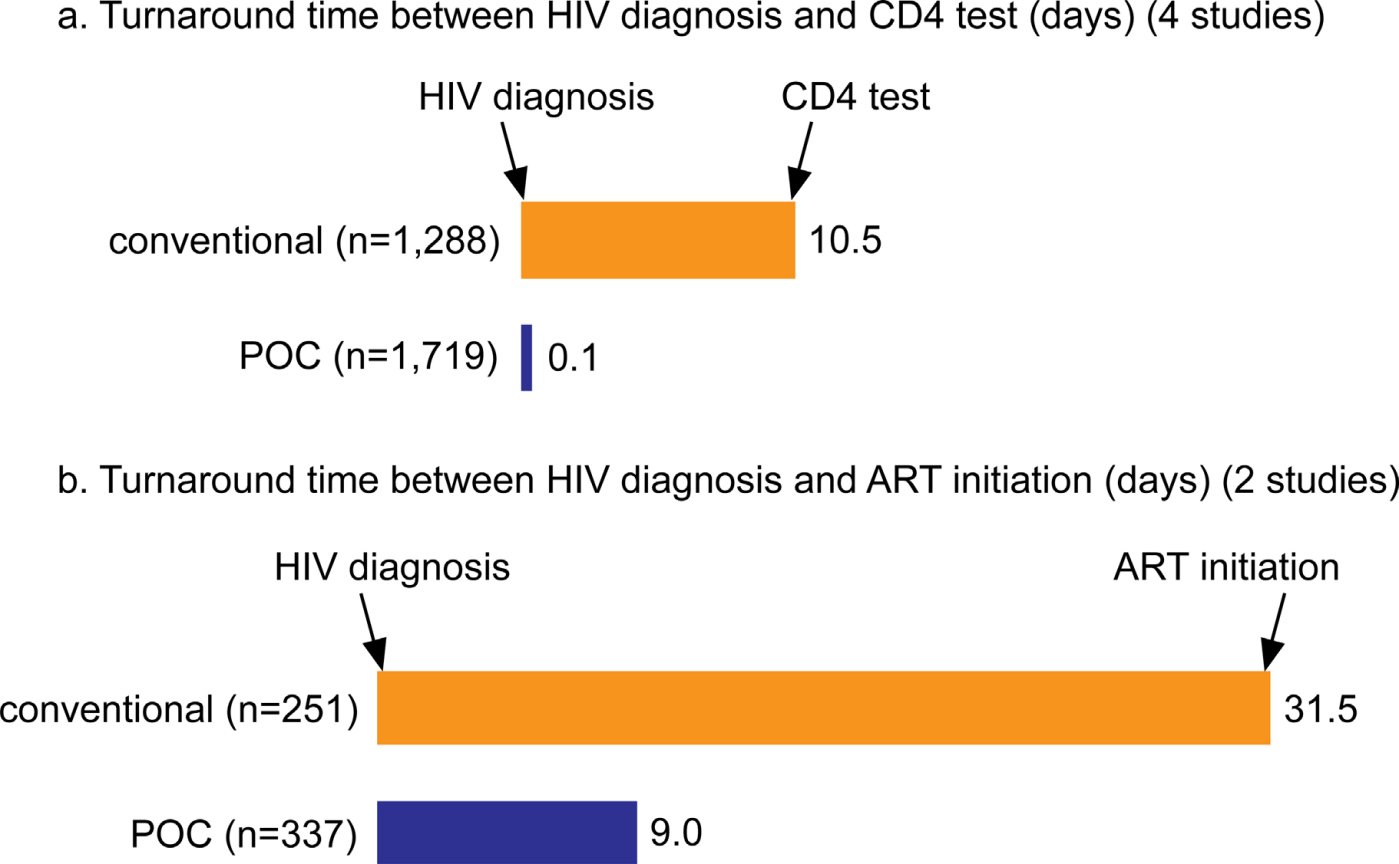
Mean turnaround times in days between two stages in the testing and treatment cascade. (a) CD4 test conducted to CD4 test result received, (b) HIV diagnosis and ART initiation. Orange bars represent the mean turnaround times for conventional laboratory-based testing, while blue bars represent the mean turnaround times for POC CD4 testing.

POC CD4 technologies reduced test turnaround times from HIV diagnosis to CD4 test and HIV diagnosis to ART initiation when compared with conventional laboratory-based testing (Fig 2). The time from HIV diagnosis to CD4 test had a pooled mean of 10.5 days with conventional laboratory-based testing. This was reduced to a pooled mean of 0.1 days with POC CD4 testing. The time from HIV diagnosis to ART initiation was reduced from a mean of 31.5 days with conventional laboratory-based testing to a mean of 9.0 days with POC CD4 testing.

### Retention along the testing and treatment cascade

**Fig 3:**
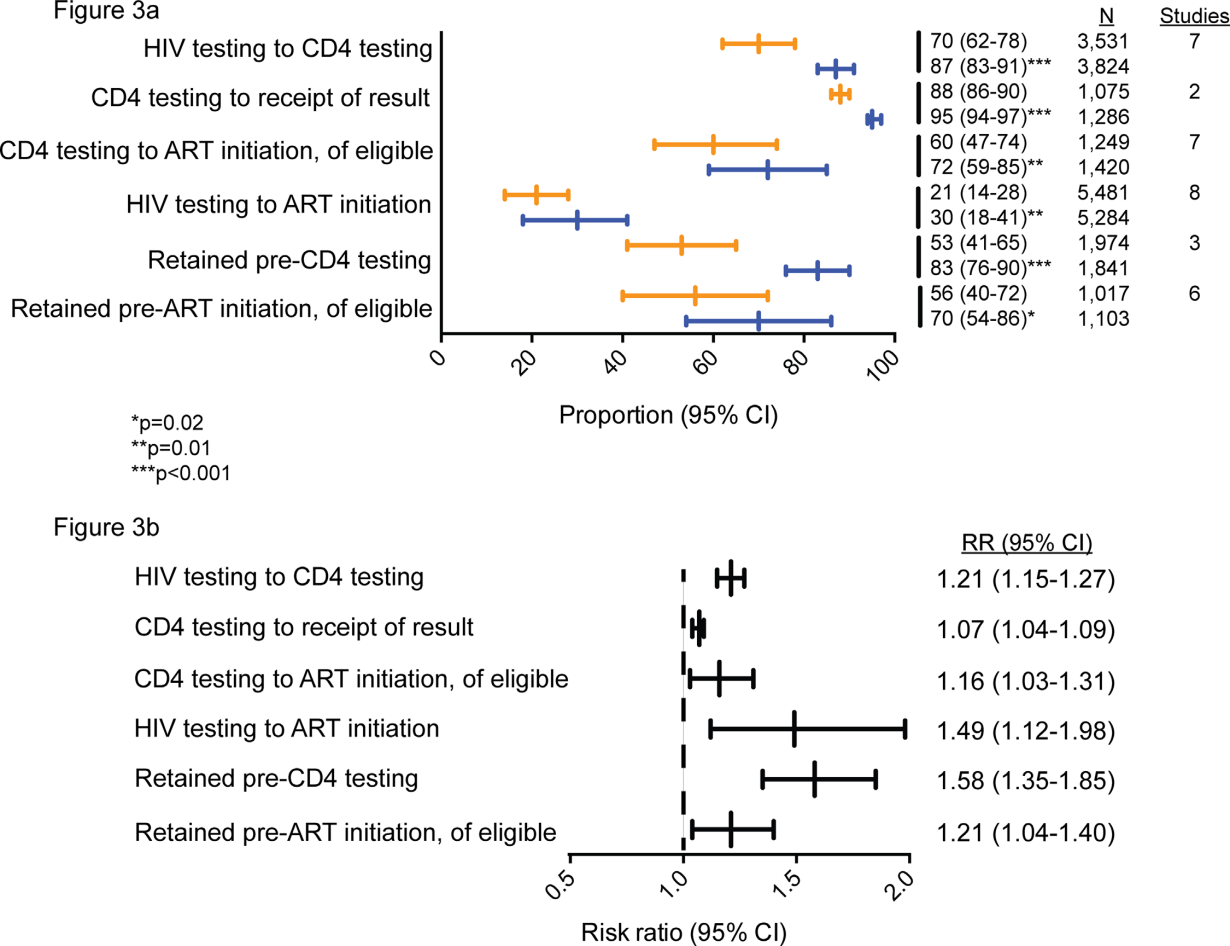
Proportions and risk ratios of patients reaching the next stage in the testing and treatment cascade. (a) Proportions of patients reaching the next stage in the testing and treatment cascade. Orange dashes and bars represent the estimated proportion of retention and 95% confidence intervals of patients receiving conventional laboratory-based testing, while blue dashes and bars represent the estimated proportion of retention and 95% confidence intervals of patients receiving POC CD4 testing. (b) Risk ratios for the likelihood of retention until the next stage in the testing and treatment cascade.

POC CD4 improved retention between several steps along the testing and treatment cascade. Pooled proportions of patients reaching the following step in the cascade were significantly higher for POC CD4 compared to conventional laboratory-based testing (Fig 3). The proportion of patients who received a CD4 test after HIV testing increased from 70% (95% CI: 62–78%) with conventional laboratory-based testing to 87% (95% CI: 83–91%) with POC CD4 testing (RR: 1.21; 95% CI: 1.15–1.27; p<0.001). Significantly more patients received their CD4 test result after POC testing (95%; 95% CI: 94–97%) compared to conventional laboratory-based testing (88%; 95% CI: 86–90%) (RR: 1.07; 95% CI: 1.04–1.19; p<0.001). Patients had a 58% higher likelihood of being retained prior to CD4 testing with POC compared to conventional laboratory-based testing, 83% (95% CI: 76–90%) versus 53% (95% CI: 41–65); RR: 1.58; 1.35–1.85; p<0.001). Additionally, significantly more patients were retained between CD4 testing and ART initiation with POC CD4 (72%; 95% CI: 59–85) compared with conventional laboratory-based testing (60%; 95% CI: 47–74) (RR: 1.16; 1.03–1.31; p = 0.01).

### Literature review of cost-effectiveness of POC CD4

Four studies looked at the cost-effectiveness of POC CD4 testing. A study from Mozambique found a 0.7-year increase in the projected life expectancy using POC CD4 compared to conventional laboratory-based testing [31]; POC CD4 was found to be cost-effective with an ICER/life year saved of $500. In a mobile unit in South Africa, an increase of 0.71 years and an ICER/life year saved of $2,400 USD was estimated to result from the inclusion of POC CD4 testing [19] as part of the overall mobile unit. Another study from South Africa focused on maternal to child transmission (MTCT) rates and observed a 0.4% reduction in transmission with the use of POC CD4 over conventional laboratory-based testing; however, this study was completed before Option B (ART for pregnant and breastfeeding women during the mother-to-child transmission risk period and maintain after cessation of breastfeeding for those eligible based on their own health) or Option B+ (lifelong ART for all pregnant and breastfeeding women living with HIV) were introduced [22,47]. Finally, a study from Malawi reported the cost per life saved with POC CD4 testing was $148.30 compared to $165.50 with conventional laboratory-based testing [28].

### Literature review of acceptability

Two studies, both from the UK, assessed the acceptability of POC CD4 testing. The first study, reported that >80% of patients found waiting 20 minutes for test results acceptable, while approximately 60% found the POC test preferable to conventional laboratory-based testing [30]. The second study found POC CD4 testing to be highly accepted compared to conventional laboratory-based testing; both new and stable patients reported significant time reductions of clinic visits [41].

## Discussion

POC CD4 technologies have been implemented in over 2,000 health care facilities in at least 70 countries around the world with large, well-established programs in several countries in sub-Saharan Africa, Southeast Asia, and Central and South America [48]. This level of scale-up has provided a good opportunity to assess the clinical impact of POC CD4 testing. Many studies have reported positive impact on turnaround times, rates of ART initiation, and loss to follow up when POC CD4 is used compared to conventional laboratory testing in the current public health approach of utilizing CD4 as a gateway to ART.

A previous systematic review of 15 published studies reported an increased likelihood of having CD4 measured (OR = 4.1), an increased likelihood of receiving a CD4 result (OR = 2.8), an increased likelihood of proceeding from CD4 to ART initiation (OR = 1.8), a reduced test turn-around time by 9 days and a reduced time from CD4 testing to receiving the result by 17 days [14]. Consistent with these previous findings, our review found that POC CD4 improved the timeliness of testing and retention of patients along the testing and treatment cascade. Time between HIV diagnosis and CD4 test and between HIV diagnosis and ART initiation were substantially reduced with the use of POC CD4 when compared to conventional laboratory-based CD4 testing. Retention along the testing and treatment cascade was significantly improved with POC CD4 testing, notably between HIV testing to CD4 testing, receipt of CD4 results, and CD4 testing and ART initiation. Finally, there was some evidence that POC CD4 was cost-effective and acceptable.

There are several limitations to this review. First, although both observational and randomized evidence was sought, most of the included studies were observational; such study designs may better reflect implementation practice, but are at higher risk of bias. All but five studies used the Alere Pima™ POC CD4 platform as the intervention. While it is not anticipated that different POC CD4 technologies with similar test times and error rates would result in variable patient impact, this could not be assessed with the limited data available. Also, participants in most studies were not consecutively recruited or failed to report the process of patient recruitment, while the rationale for facility selection was not indicated or not randomized in most studies. The possibility exists that some studies may suffer from patient or facility selection bias. Another limitation is that the applicability of these findings beyond Africa is unclear as almost all studies were carried out in Africa, despite at least 70 countries and many resource-limited countries having adopted POC CD4 technologies [48]. Finally, the findings were heterogeneous across studies (S3 Fig). Little detail was provided on the function of the conventional testing systems; however, the variable findings were likely due to significantly diverse quality of the conventional laboratories and related systems across different settings and countries included in the analysis.

The cost-effectiveness of POC CD4 is well-defined; however, affordability analyses with clear, detailed costing would allow stakeholders to carefully consider the adoption and scale-up of POC CD4 in light of changing guidelines and restricted budgets. Also, operational implementation guidance and tools based on best practice experiences would be valuable to inform use of these technologies. Furthermore, guidelines and optimal models for ensuring high quality, reliable test results for both rapid and point of care tests would be valuable.

Recent WHO Consolidated ART guidelines recommend treating all patients living with HIV, regardless of CD4 count, and using viral load testing to monitor patients on ART [5,47]. CD4 testing is, however, recommended for monitoring opportunistic infections of unstable or sick patients. The frequency and utility of CD4 testing may, therefore, decline in the future; however, many national programs in high HIV burden countries have only begun implementing routine viral load testing and few have adopted the treat all recommendation into national policies.

Widespread uptake and decentralization of rapid diagnostic tests for major public health challenges, including HIV, tuberculosis and malaria, have led to significant gains in health provision and greater access to treatment, particularly in resource-limited settings [49]. While centralized laboratory networks are important for high throughput sample processing and quality control, they are unable to provide access to same day testing services to all patients in need. Also, sample transportation networks can be weak in many resource-limited settings resulting in long test turnaround times. POC tests are rapid, easy to use, and significantly improve retention of patients along the testing and treatment cascade and reduce the time from diagnosis to treatment compared to conventional laboratory-based testing. For POC tests to contribute maximally to laboratory networks, however, supportive systems such as supply chain, training and monitoring, quality assurance, service and maintenance, and data management must be built into the testing network and strengthened for improved patient care.

## Supporting Information

S1 Fig Completed 2009 PRISMA Checklist. (DOC)

S2 Fig EMBASE Search Strategy. (DOCX)

S3 Fig Forest plots of each outcome analyzed. (TIF)
