# Supplementary material for: POC CD4 Testing Improves Linkage to HIV Care and Timeliness of ART Initiation in a Public Health Approach: A Systematic Review and Meta-Analysis
Source: PLoS One. 2016 May 13;11(5):e0155256. doi: 10.1371/journal.pone.0155256 (PMC4866695; doi:10.1371/journal.pone.0155256)
Supplement: S2 Fig — (DOCX) [file pone.0155256.s002.docx]

**EMBASE Search Strategy**

(HIV OR “HIV infections” OR hiv OR hiv-1 OR hiv-2 OR hiv1 OR hiv2 OR “hiv infect” OR “hiv infected” OR “hiv infection” OR “human immunodeficiency virus” OR “human immunedeficiency virus” OR “human immuno-deficiency virus” OR “human immune-deficiency virus” OR “acquired immunodeficiency syndrome” OR “acquired immunedeficiency syndrome” OR “acquired immuno-deficiency syndrome” OR “acquired immune-deficiency syndrome”)

AND

(POC OR POCT OR “point of care” OR point-of-care OR rapid OR portable OR mobile OR remote OR Alere OR Pima OR “Becton Dickinson” OR BD OR FACSPresto OR Daktari OR mBio OR Omega OR Burnet OR Partec OR PointCare OR Guava OR Millipore OR Muse OR Zyomyx OR MyT4 OR ChipCare)

AND

(CD4 OR CD4+ OR “T cell” OR “helper cell” OR “cluster of differentiation 4”)

AND

(LTFU OR retention OR linkage OR attrition OR TAT OR “turnaround time” OR “time from test to result” OR “result returned” OR “results returned” OR “time to care” OR “time to refer” OR “time to referral” OR “time to ART” OR time to drug* OR “TAT to ART” OR “adverse event” OR “adverse events” OR morbidity OR mortality OR care OR follow-up OR “follow up” OR “result notification” OR cost OR cost-effectiveness OR DALY OR ICER OR affordability OR affordable OR feasibility OR feasible OR acceptability OR acceptable OR confidential OR confidentiality OR safety OR accuracy OR performance OR “technical evaluation “)

AND

Publication date after January 1, 2005
