# Supplementary figures and images for: POC CD4 Testing Improves Linkage to HIV Care and Timeliness of ART Initiation in a Public Health Approach: A Systematic Review and Meta-Analysis
Source: PLoS One. 2016 May 13;11(5):e0155256. doi: 10.1371/journal.pone.0155256 (PMC4866695; doi:10.1371/journal.pone.0155256)

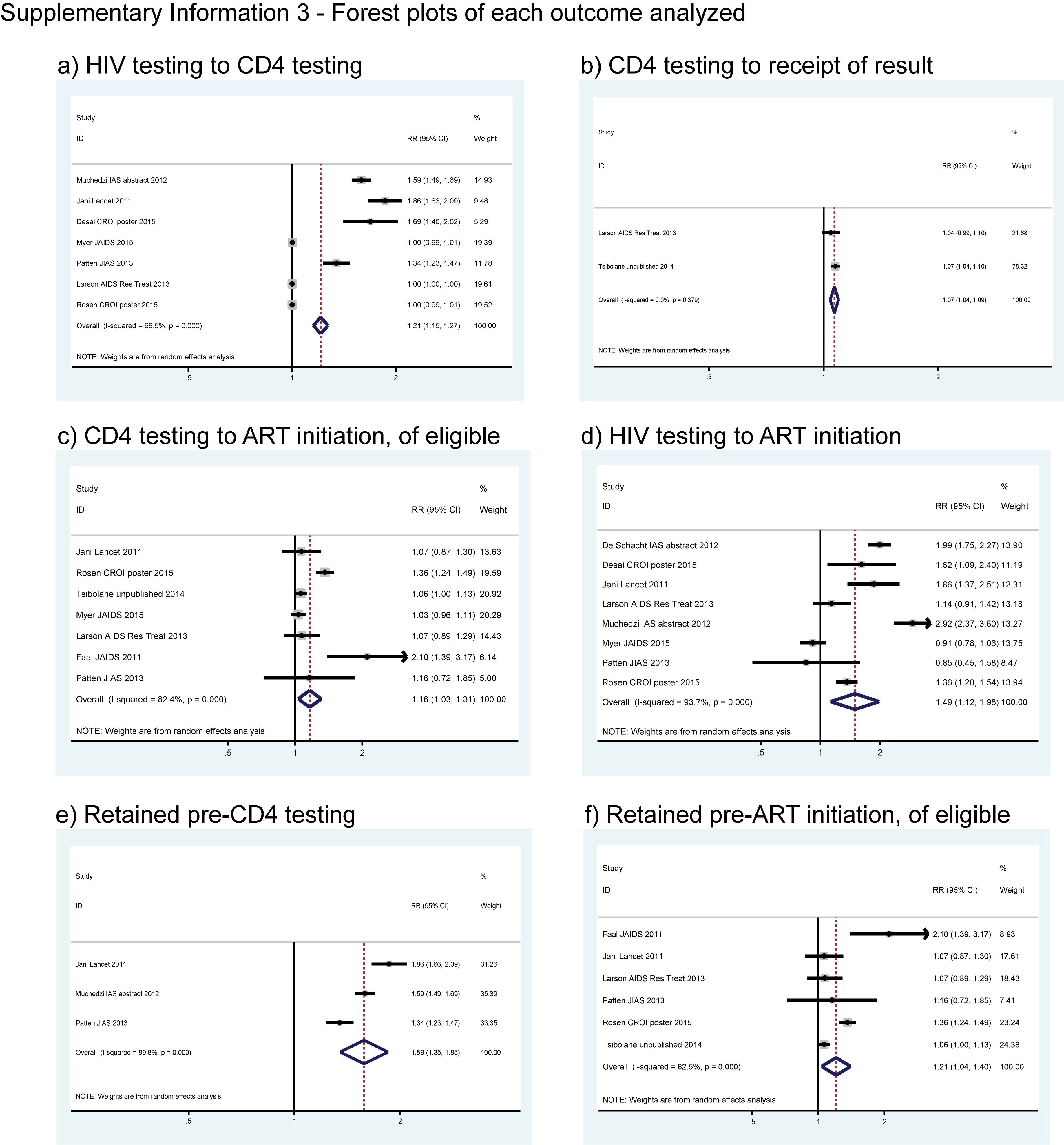

Supplement: S3 Fig — (TIF) [file pone.0155256.s003.tif]
